# Magnetic field exposure and long-term survival among children with leukaemia

**DOI:** 10.1038/sj.bjc.6602916

**Published:** 2006-01-10

**Authors:** D E Foliart, B H Pollock, G Mezei, R Iriye, J M Silva, K L Ebi, L Kheifets, M P Link, R Kavet

**Affiliations:** 1 Public Health Institute, 555 12th St, Oakland, CA 94607, USA; 2 Center for Epidemiology and Biostatistics, University of Texas Health Science Center, 7703 Floyd Curl Drive, San Antonio, TX 78229, USA; 3 Electric Power Research Institute (EPRI), 3412 Hillview Avenue, Palo Alto, CA 94304, USA; 4 Enertech Consultants, 300 Orchard City Drive, Suite 132, Campbell, CA 95008, USA; 5 Exponent Health Sciences Group, 1800 Diagonal Road, Suite 300, Alexandria, VA 22314, USA; 6 Department of Epidemiology, School of Public Health, University of California, Los Angeles, 650 Charles E Young Drive South, Los Angeles, CA 90095, USA; 7 Department of Pediatrics, Stanford University School of Medicine, 300 Pasteur Drive, Stanford, CA 94305, USA

**Keywords:** electromagnetic fields, leukaemia, lymphoblastic leukaemia, acute childhood leukaemia

## Abstract

We examined the association between magnetic field (MF) exposure and survival among children with acute lymphoblastic leukaemia (ALL) treated at 51 Pediatric Oncology Group centres between 1996 and 2001. Of 1672 potentially eligible children under treatment, 482 (29%) participated and personal 24-h MF measurements were obtained from 412 participants. A total of 386 children with ALL and 361 with B-precursor ALL were included in the analysis of event-free survival (time from diagnosis to first treatment failure, relapse, secondary malignancy, or death) and overall survival. After adjustment for risk group and socioeconomic status, the event-free survival hazard ratio (HR) for children with measurements ⩾0.3 *μ*T was 1.9 (95% confidence interval (CI) 0.8, 4.9), compared to <0.1 *μ*T. For survival, elevated HRs were found for children exposed to ⩾0.3 *μ*T (multivariate HR=4.5, 95% CI 1.5–13.8) but based on only four deaths among 19 children. While risk was increased among children with exposures above 0.3 *μ*T, the small numbers limited inferences for this finding.

Two pooled analyses have reported a positive association between childhood leukaemia incidence and residential magnetic fields (MFs) in the upper tail of the exposure distribution (Ahlbom *et al*, 2000; Greenland *et al*, 2000). However, *in vitro* studies and *in vivo* animal experiments have not produced evidence of adverse effects at or near the MF levels associated with residential environments, nor has a biophysical basis for such effects been established (NIEHS, 1998; McCann *et al*, 2000). While earlier studies have examined potential effects of MF exposure on leukaemia incidence, MF research has not focused on the progress of disease. We have examined our hypothesis that if environmental exposure to MF influences leukaemia blast cells following tumour initiation, effects on relapse and survival in newly diagnosed acute lymphoblastic leukaemia (ALL) may be evident.

## MATERIALS AND METHODS

### Study subjects

Eligibility criteria included a diagnosis of B-precursor, T-cell, or B-cell ALL within the previous 12 weeks, age between 1 and 15 years, enrolment into a Pediatric Oncology Group (POG) treatment protocol at a participating centre, and an adult family member who speaks English, Spanish, or French. Pediatric Oncology Group was a National Cancer Institute-sponsored consortium that represented approximately half of all centres that treated childhood cancer in North America. The study protocol was approved by the institutional review boards of the Public Health Institute and each participating treatment centre.

Health professionals at POG centres introduced the study to eligible children and their families, and obtained written informed consent from interested families. During the accrual period of September 1996 to January 2001, 1672 children were being treated on therapeutic protocols at the 51 participating POG centres. Twenty nine per cent (482) of children enrolled into our study. The study group was similar in gender and age to the cohort of eligible children, although percentages of children by race/ethnicity differed: white children 72% (cohort) *vs* 61% (potentially eligible), Hispanic children 15 *vs* 21%, African-American children 7 *vs* 9%.

Families were contacted within 14 days of an agreement to participate by staff at the Public Health Institute for administration of a structured telephone interview and explanation of the exposure assessment process. The interview included questions regarding sociodemographic characteristics (parental education and family annual income) and residential history. Ninety-eight per cent (471 out of 482) of families completed the telephone interview.

### Data collection

#### Magnetic field exposure assessment

Magnetic field exposure assessment was initiated after the child completed the initial induction therapy (usually 4 weeks from the start of therapy) and while the child was undergoing consolidation (intensification) therapy as an outpatient. The EMDEX Lite meter (Enertech Consultants Campbell, CA, USA), with pictorial and written instructions (English, Spanish, or French), was forwarded to families and returned by mail. Personal, 24-h MF exposures were monitored prospectively, with the first measurement taken shortly after enrolment and later measurements taken at the beginning of the second and third year after enrolment. The exposure protocol and results of demographic and serial MF exposure monitoring have been previously reported (Foliart *et al*, 2001, 2002).

A higher-than-expected percentage of families lost or never returned the meter (8%). Some families were reluctant to use the meter or had difficultly following the pictorial instructions, with 5% returning the meter with invalid or no data recorded. Based on follow-up telephone conversations with most of the 70 families who did not return valid first-year readings, it was clear that the diagnosis of leukaemia produced tremendous family stress and disruption, making it difficult to complete the exposure assessment protocol.

Completion of the exposure assessment protocol with return of viable data decreased during annual serial monitoring: 412 children completed the first monitoring, 304 completed a second measurement, and 134 completed a third measurement. The mean time-weighted average (TWA) (0.11–0.13 *μ*T) and mean geometric mean (GM) (0.073–0.082 *μ*T) were similar across the three measurement years (Foliart *et al*, 2002). Owing to the limited number of second- and third-year measurements, only the first year 24-h results were used in our analyses.

*A priori*, MF exposures were classified in a variety of ways, including TWA, GM, and two estimates of field stability: rate of change metric (RCM) and rate of change metric standardised (RCMS) (Burch *et al*, 1998). The metrics with the highest year-to-year correlation were TWA and GM. Rate of change metric and RCMS were poorly correlated over time (Foliart *et al*, 2002). Our final analyses used 24-h exposure classified by TWA and GM.

#### Clinical data

At the time of diagnosis, all children had blood and bone marrow samples sent to a central reference laboratory at the University of Alabama, Birmingham, for cytogenetic analysis. For central assignment to their therapeutic regimen, children were stratified by immunophenotype and clinical prognostic factors by the POG Statistical Office at the University of Florida (Gainesville, FL, USA). For the commonest immunophenotype, B-precursor ALL, children were enrolled into one of three POG protocols based on prognostic factors at diagnosis predicting low, standard, or high risk of treatment failure (Borowitz *et al*, 1993). For children with B-precursor ALL enrolled before 2000 (330 out of 361 children), the National Cancer Institute (NCI) risk group criteria were used to determine the risk status, based on initial white blood cell count and age at diagnosis (Smith *et al*, 1996). In 2000, DNA Index and the presence of trisomies 4 and 10 were added to the risk stratification. While the criteria used to identify a child's risk were refined, children in a given risk stratum received similar treatment protocols over the course of the study. Protocols for T- and B-cell ALL did not change over the course of the enrolment period.

### Data analysis

Of the 482 enrolled children, 412 (85%) completed the questionnaire and first-year MF assessment protocol and 386 (80%) were included in the analyses. The 26 ineligible children included 13 with missing data regarding response to induction therapy, 10 who did not enrol on a standardised treatment protocol, two with no follow-up data, and one whose diagnosis was later confirmed not to be ALL.

The primary outcome for this study was event-free survival, with additional analyses restricted to overall survival. Kaplan–Meier curves (Kaplan and Meier; 1958) with log rank tests (Peto *et al*, 1977) were used to examine the relationship between MF exposure (TWA<0.1, 0.1–0.19, 0.2–0.29, >0.3 *μ*T) and outcome. Event-free survival was defined as the interval of time from diagnosis to the date of last contact or to any of the following events that occurred: failure to attain a complete remission after induction therapy (Roberts *et al*, 1997), leukaemia relapse, secondary malignancy, or death from any cause. Removal for bone marrow transplantation was considered a censoring event (Pollock *et al*, 2000). Event-free survival was tracked through December 2004. Analyses were performed for all subjects combined and separately for B-precursor and T-cell ALL patients. Analysis of B-precursor ALL patients was adjusted by the NCI risk group criteria.

Multivariate analyses were conducted using Cox proportional hazards regression (Cox, 1972). The association between MF exposure and outcome, including analyses of trend across MF strata, as well as the association between other covariates and outcome, was estimated as the hazards ratio (HR) with 95% confidence interval (CI). Subjects with MF exposure <0.1 *μ*T constituted the reference group. Regression analyses were adjusted for a number of potential confounders and effect modifiers: NCI risk group, race/ethnicity, immunophenotype, and socioeconomic status (SES). Low SES was defined as annual family income <$40 000 and both parents with less than a college degree.

A number of other covariates were assessed in the secondary analyses. These included DNA Index, platelet count at diagnosis, presence of central nervous system involvement at diagnosis, trisomies 4 and 10, trisomy 21, trisomy 8, and several relatively rare cytogenetic translocations including t(9;22), t(4;11), and t(1;19). We examined the impact of these less common prognostic factors by individually adding them to a Cox regression model that included a primary MF exposure category and the NCI risk group.

## RESULTS

Of the 386 children included in the analysis, 71% were less than 6 years old. Boys slightly outnumbered girls (52%). Table 1 presents details of race/ethnicity and SES. Most children (75%) were white; 41% were classified as having low SES. Annual family income, paternal education, and, to a lesser extent, maternal education varied widely by race and ethnic group (Table 1).

Most children completed the MF monitoring session shortly after diagnosis: 48% (186) within 2 months following diagnosis and 86% (332) within 4 months. The mean TWA was similar to that in other North American studies: 0.1 *μ*T, with a 95th percentile value of 0.3 *μ*T (Kaune and Zaffanella, 1994; Linet *et al*, 1997; Deadman *et al*, 1999).

Table 2 summarises the MF exposure findings. Only 19 children (5%) had a TWA⩾0.3 *μ*T and 14 (4%) had a GM⩾0.3 *μ*T. A higher percentage of non-white compared with white children (7.3 *vs* 4.1%) had TWA exposures ⩾0.3 *μ*T (odds ratio=1.8, 95% CI 0.70, 4.77).

The median duration of follow-up among survivors was 5.07 years. There were 73 failure events among all children and 70 failure events among the 361 children with B-precursor ALL. There was a total of 30 deaths, of which 28 occurred among children with B-precursor ALL. In only one child was death the first failure event. For the 29 other children, the initial failure event was relapse (*n*=25) or secondary malignancy (*n*=4).

**Figure 1:**
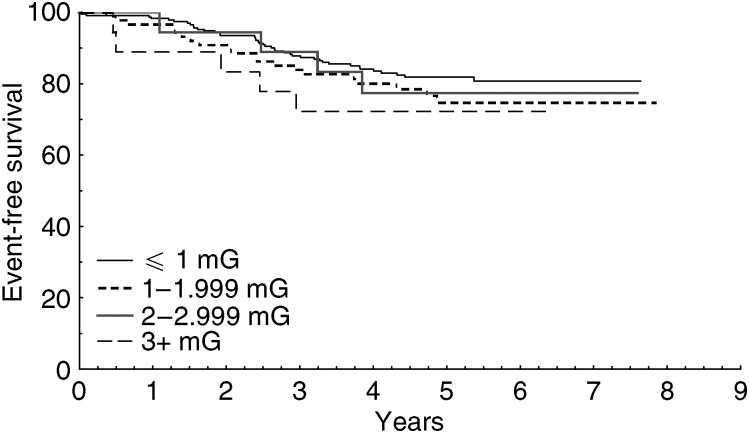
Kaplan–Meier estimates for event-free survival among children with B-precursor ALL, stratified by 24-h TWA MF exposure (log rank test *P*=0.054).

Figure 1 presents the Kaplan–Meier estimate of event-free survival for children with B-precursor ALL, stratified by 24-h TWA MF exposures (log rank test *P*=0.54). Estimates using GM were similar and are not presented. Owing to small numbers, figures for total survival and T-cell ALL are not presented.

Cox proportional hazards regression analyses of survival are presented in Table 3. No statistically significant trend was noted between increasing exposure to MF and poorer event-free survival (*P*=0.5). Five failures were observed among children exposed to ⩾0.3 *μ*T, four due to relapse and one due to secondary malignancy (four died during follow-up). Hazard ratios for children in the highest exposure category of ⩾0.3 *μ*T were increased in both univariate and multivariate analyses. In univariate analysis, the event-free survival HR for exposure ⩾0.3 *μ*T was 1.66, 95% CI 0.66, 4.18. Multivariate analyses of B-precursor ALL, adjusted for age at diagnosis and initial white blood cell count (NCI risk group) and SES, reported an HR for exposure ⩾0.3 *μ*T of 1.92, 95% CI 0.75, 4.90. Of the five failures in this exposure group, two occurred among the 11 white children and three occurred among the seven non-white children. Although failures were more common among non-white children, small numbers prevented us from adequately examining race-specific risks.

For overall survival, HRs were significantly elevated among children exposed to ⩾0.3 *μ*T in both univariate and multivariate analyses, based on four deaths (two in white children and two in non-white children). In univariate analysis, the HR was 3.39, 95% CI 1.14, 10.06; in multivariate analysis, the HR was 4.53, 95% CI 1.49, 13.76. In the multivariate analysis, there was a marginal trend (*P*=0.06) between increasing exposure category and deaths, although the number of deaths was small, with only one observed among the 19 children exposed to ⩾0.2–0.29 *μ*T.

As expected, children in the higher NCI risk group were at an increased risk of poor outcome for both event-free survival and total survival. Socioeconomic status was not associated with outcome. No significant first-order interaction terms were observed between MF exposure and the following covariates: DNA Index, platelet count at diagnosis, presence of central nervous system blast cells at diagnosis, and presence of translocations or trisomies (data not presented).

## DISCUSSION

Although other studies have investigated MF exposure as a risk factor for incident ALL, this is the first study to address MF exposure and long-term survival among children with ALL. Strengths of the study include its prospective cohort design, use of centralised review of malignancy and biologic markers, inclusion of children from a wide geographic area, personal MF dosimetry, and the length and completeness of follow-up. Because failure events are most likely to occur within the first 5 years following diagnosis (Pollock *et al*, 2000), the study had an adequate duration of follow-up to capture most outcome events.

Three limitations of our study are noteworthy. As has been seen in earlier studies, only 5% of the cohort (19 children) had a TWA above 0.3 *μ*T (Kaune and Zaffanella, 1994; Linet *et al*, 1997; Deadman *et al*, 1999). Thus, we had limited ability to evaluate outcome among children exposed to more than 0.3 *μ*T and could perform no meaningful analyses above 0.4 *μ*T. Secondly, less than one-third of potentially eligible children enrolled into our study, with lower participation rates among non-white children. The limited number of non-white children prevented meaningful multivariate analyses by racial/ethnic subgroups. Finally, our survival analyses were based on first-year MF exposure assessments. In an analysis of the subset of 304 children with two or more annual measurements, we found that first-year GM and TWA can serve as an estimate of exposure among residentially stable children (Foliart *et al*, 2002). However, a single measurement was less useful among children who changed residences. Twelve per cent of children with first-year measurements moved during the exposure assessment protocol. The total number of children who changed residences during the entire course of follow-up is unknown. The single assessment of MF exposure in residentially mobile children may not be an adequate surrogate of MF exposure over the study's extended follow-up period.

The focus of our study was the possible role of MF exposure as an independent predictor of event-free and overall survival. The number of failures was small: four among children exposed to ⩾0.2–0.29 *μ*T and five among children exposed to ⩾0.3 *μ*T. For overall survival, the HRs were significantly elevated among children exposed to ⩾0.3 *μ*T for both univariate and multivariate analyses. The multivariate HR (4.5) was based on only four deaths, yielding a wide CI (1.5, 13.8). No consistent or statistically significant trend was noted between increasing exposure to MF and event-free survival or risk of death. Although we report poorer survival among children with the highest MF exposure category, clinical inferences are limited, with results possibly attributable to chance alone. Independent confirmation is needed, as our study is the first to look at relapse and survival and thus our findings can be viewed only as hypothesis generating.

## Figures and Tables

**Table 1:** Race, ethnicity, and socioeconomic status of cohort^a^

		**Socioeconomic status**		**Annual income**		**Maternal education**		**Paternal education**	
	**No. (%)**	**High**	**Low**	**>$40 000**	**<$40 000**	**Some college**	**⩽High school**	**Some college**	**⩽High school**
White	290 (75)	239 (86)	39 (14)	150 (53)	132 (47)	196 (69)	88 (31)	182 (65)	98 (35)
African American	20 (5)	11 (73)	4 (27)	1 (5)	17 (94)	14 (74)	5 (26)	4 (27)	11 (73)
Hispanic	50 (13)	24 (56)	19 (44)	11 (22)	37 (77)	19 (39)	30 (61)	12 (27)	32 (73)
Native American	2 (0.5)	2 (100)	0	0	2 (100)	1 (50)	1 (50)	1 (50)	1 (50)
Asian	11 (3)	10 (100)	0	9 (90)	1 (10)	11 (100)	0	10 (91)	1 (9)
Hawaiian	9 (2)	6 (75)	2 (25)	3 (33)	6 (67)	5 (56)	4 (44)	4 (50)	4 (50)
Other	4 (1)	2 (50)	2 (50)	2 (50)	2 (50)	2 (50)	2 (50)	2 (50)	2 (50)
Response missing	0	26		13		8		22	

a Per cent in parentheses, based on total with available information. A total of 386 families completed the telephone questionnaire, but answers to some queries were not known by respondents.

**Table 2:** Summary of 24-h time-weighted average (TWA) and geometric mean (GM) magnetic field exposure results

	**TWA**		**GM**	
**Exposure (*μ*T)**	**Frequency**	**%**	**Frequency**	**%**
<0.1	251	65	294	76
0.1–0.19	95	25	67	17
0.2–0.29	21	5	11	3
0.3–0.39	7	2	10	3
0.4–0.49	7	2	4	1
0.5–0.59	1	0.3	0	0
⩾0.6	4	1	0	0
Total	386		386	

**Table 3:** Association of magnetic field exposure and outcome: Cox proportional hazards regression analyses

	**Event-free survival**						**Survival**					
**Variable**	**No. of cases**	**No. failed^a^**	**HR**	**95% HR CI^b^**		***P* c**	**No. of cases**	**No. failed**	**HR**	**95% HR CI**		**P^c^**
*Univariate analysis*	386	73				0.4	386	30				0.2
<0.1 *μ*T	251	42	1.00				251	17	1.00			
0.1–0.19 *μ*T	95	22	1.47	0.88	2.46		95	8	1.27	0.55	2.93	
0.2–0.29 *μ*T	21	4	1.15	0.41	3.20		21	1	0.68	0.09	5.13	
⩾0.3 *μ*T	19	5	1.66	0.66	4.18		19	4	3.39	1.14	10.06	
												
*Multivariate analysis*												
B-precursor^d^	361	70				0.5	361	28				0.06
NCI risk group			1.76	1.01	2.88				2.78	1.29	5.96	
SES			0.98	0.52	1.83				1.61	0.68	3.82	
<0.1 *μ*T	235	41	1.00				235	16	1.00			
0.1–0.19 *μ*T	89	20	1.25	0.72	2.19		89	7	1.16	0.47	2.84	
0.2–0.29 *μ*T	19	4	1.32	0.47	3.71		19	1	0.85	0.11	6.40	
⩾0.3 *μ*T	18	5	1.92	0.75	4.90		18	4	4.53	1.49	13.76	

a Failure events defined as failure to attain complete response during induction therapy, leukaemia relapse, secondary cancer, or death.

b HR=hazard ratio; CI=confidence interval.

c Test of trend for time-weighted average magnetic field exposure level.

d Adjusted for National Cancer Institute (NCI) risk group and socioeconomic status (SES).
